# Urban Expansion and Butterfly Diversity: The Synergistic Effects of Impervious Surface and Vegetation Cover

**DOI:** 10.3390/insects17050482

**Published:** 2026-05-08

**Authors:** Jinlu Zhang, Zhouyang Liao, Xuemei Shen, Mi Zhu, Xiaozhang Chen, Zhibo Feng, Yuan Zhang

**Affiliations:** 1 Key Laboratory of Forest Disaster Warning and Control of Yunnan Province, Southwest Forestry University, Kunming 650224, China; jinlu_zhang@swfu.edu.cn (J.Z.); zhouyang_liao@swfu.edu.cn (Z.L.); xuemei_shen@swfu.edu.cn (X.S.); mi_zhu@swfu.edu.cn (M.Z.); xiaozhang_chen@swfu.edu.cn (X.C.); zhibo_feng@swfu.edu.cn (Z.F.); 2 College of Forestry, Southwest Forestry University, Kunming 650224, China

**Keywords:** urbanization, butterfly diversity, impervious surface, vegetation cover

## Abstract

Under the background of global urbanization, the impacts of urban expansion on biodiversity are becoming increasingly significant. Butterflies, as key ecological indicator species and important pollinators, are crucial for studying population dynamics and their driving factors in order to support biodiversity conservation and environmental governance. Butterflies also have important ecological value in urban biodiversity conservation and landscape maintenance. Currently, in many countries, especially developing countries, impervious surfaces and vegetation cover are undergoing significant changes as urbanization intensifies. Existing studies have begun to focus on the effects of these changes on biodiversity conservation. However, most studies primarily focus on the single effects of these two factors on biodiversity, while systematic assessments of their potential interactive effects remain limited. In this study, monthly butterfly population monitoring was conducted in Kunming, China, for two consecutive years. The study aimed to clarify the composition of butterfly diversity in Kunming and to explore the effects of impervious surfaces and vegetation cover on butterfly species richness, abundance, Shannon index, Simpson index, and community structure. The results showed that impervious surface was the primary driving factor influencing both butterfly richness and abundance, while the influence of vegetation cover was relatively limited. The Shannon and Simpson indices were not significantly influenced by either environmental factor. Environmental filtering caused by impervious surfaces and vegetation cover drove species turnover processes. Consequently, butterfly communities exhibited the highest heterogeneity in areas characterized by low impervious surfaces and high vegetation cover. In contrast, areas with high impervious surfaces and low vegetation cover tended to show a pattern of community homogenization. Urban planning should balance the control of impervious surface proportions with the appropriate enhancement of vegetation cover and plant diversity. Such measures may mitigate the negative impacts of intensified urbanization on butterfly diversity to some extent.

## Simple Summary

Urban expansion presents substantial challenges to biodiversity, and butterflies serve as valuable indicators of these environmental alterations. We carried out a two-year monthly butterfly monitoring program in Kunming, China, to investigate how these factors affect butterfly species richness, abundance, Shannon index, Simpson index, and community structure. We discovered that impervious surface is the principal factor determining butterfly richness and abundance, with vegetation cover playing a secondary part. The Shannon and Simpson indices were not significantly influenced by either environmental factor. Collectively, these factors trigger changes in butterfly species composition: areas featuring low impervious surfaces and high vegetation cover possess the most diverse butterfly communities, whereas areas with high impervious surfaces and low vegetation cover have homogeneous and simplified butterfly groups. These findings imply that urban planning strategies which restrict impervious surface while appropriately increasing vegetation cover can assist in alleviating the adverse impacts of urbanization on butterfly diversity.

## 1. Introduction

With the rapid development of the global economy and the acceleration of urbanization, a series of environmental changes induced by urban expansion, such as the increase in impervious surface, the intensification of urban heat island effects, and the reduction of vegetation cover, have expedited the loss and degradation of natural habitats, presenting severe threats to biodiversity [1,2,3]. As a crucial component of biodiversity, insects are highly sensitive to urbanization. In the past few decades, global insect diversity has been experiencing a significant decline, and urbanization is regarded as one of the primary drivers of this biodiversity loss [4]. To address the environmental pressures imposed by urbanization, a growing number of countries have acknowledged that well-conceived urban planning and construction can play a significant role in biodiversity conservation. Measures like enhancing vegetation cover and expanding urban green space can not only enhance the ecological environment quality but also contribute to biodiversity conservation [5,6,7]. For instance, Singapore has substantially expanded the area of urban green space and notably enhanced vegetation cover via the “Garden City” program, thereby offering new habitats for numerous insects [8]. These ecologically-oriented development actions have also given rise to the emergence of diverse novel artificial or semi-artificial ecosystems during the process of urbanization, including urban parks, roadside green belts, rooftop gardens, and private gardens [9]. Studies have demonstrated that despite the relatively limited area of urban green space, it can still offer alternative habitats for certain species, such as butterflies, and in some instances even act as refuges for endangered species [10,11]. From a social perspective, urban biodiversity sustained by green spaces enhances residents’ well-being, supports environmental education and citizen science, and, through systematic educational actions and public engagement, promotes the conservation of critical habitats and ecological corridors while providing sustained social impetus for long-term urban biodiversity protection [12]. Butterflies, being a common urban insect taxon and flower visitors, are widely distributed in urban green spaces and have increasingly become a focal group for ecological research in recent years [13].

Butterflies are widely recognized as crucial indicator taxa for evaluating ecological changes in urban environments because of their high sensitivity to environmental factors like microclimate and vegetation structure [14]. Monitoring data from recent decades indicate that global butterfly diversity has shown a significant overall decline [15,16]. Long-term butterfly monitoring in Europe shows that the total abundance of butterflies in some countries has declined by approximately 50% [17]. About 37% of the native butterfly species in the UK have either become extinct or are endangered [18]. In North America, the population of *Danaus plexippus* declined by approximately 97% from the 1980s to the mid-2010s [19], while a recent nationwide analysis reported a 22% decline in overall butterfly abundance across the contiguous United States between 2000 and 2020 [20]. Moreover, the local extinction rate between 1992 and 2010 reached as high as 81% [21]. Tropical regions have also experienced significant butterfly declines. As of 2017, Singapore had lost 153 butterfly species, accounting for about 32% of the total recorded species [22]. Although the decline of butterfly diversity has become a global consensus, most existing studies have concentrated on natural or semi-natural habitats. The systematic understanding of butterfly community diversity and its driving mechanisms in the context of urbanization is still limited [23]. This knowledge gap is particularly evident in many developing countries where urbanization is advancing rapidly. Therefore, exploring the spatial patterns of butterfly diversity and their driving factors in urban settings is crucial for comprehending the mechanisms that sustain butterfly diversity under rapid urbanization and for optimizing urban ecological planning and management.

In the context of urbanization, many natural habitats, such as grasslands and forests, are gradually being replaced by artificial infrastructure, including buildings and roads. This replacement directly reduces the amount of habitat available to butterflies and simultaneously alters habitat quality, resource distribution, and landscape connectivity [24,25,26]. As an important indicator of urbanization intensity, impervious surface has been widely recognized as a key factor influencing biodiversity [27]. Firstly, the expansion of impervious surfaces intensifies the urban heat island effect and can significantly elevate the mean daytime temperatures at local scales. Such microclimatic alterations may surpass the physiological tolerance of butterflies and ultimately result in a decline in their diversity [28]. Moreover, urbanization can reduce the availability and diversity of larval host plants and adult nectar resources, thereby limiting larval development, adult foraging opportunities, and the persistence of butterfly populations in urban green spaces [29]. Impervious surfaces may also affect butterflies by altering the permeability of the urban matrix and restricting dispersal among habitat patches. Roads, buildings, and other artificial structures increase landscape resistance and isolate urban green spaces. Such changes may consequently limit species dispersal and habitat connectivity, particularly for species characterized by low mobility, small body size, or high habitat specialization [30]. Other studies focusing on plant cover in urban parks and similar green spaces suggest that butterfly communities dominated by large and highly mobile species can effectively utilize urban green areas and adapt to urban environments. This finding implies that human modifications to vegetation cover in some cities may partly mitigate ecological stresses caused by urbanization [13,31]. Urban vegetation can provide nectar resources for adults, host plants for larvae, and diverse microhabitats required by different functional groups [32,33]. A more complex vegetation structure, including herbaceous layers, shrubs, tree cover, and flowering plants, generally increases resource heterogeneity and can support higher butterfly abundance and diversity. Vegetation can also regulate microclimates through transpiration, effectively alleviating the physiological stress imposed on butterflies by the urban heat island effect [34]. At the landscape scale, connected vegetation patches, street-tree networks and greenways may facilitate butterfly dispersal among habitat patches, reduce isolation effects, and enhance the long-term persistence of urban populations [23,33]. Therefore, vegetation cover does not merely represent the amount of green space; rather, it reflects a combination of resource availability, habitat complexity, microclimatic buffering, and dispersal pathways. Overall, existing studies demonstrate that the factors influencing butterfly diversity during urbanization are intricate, and not all urban environmental factors inevitably have negative effects on butterfly communities. This implies that impervious surface and vegetation cover might be two crucial factors shaping butterfly diversity and its spatial distribution patterns in urban landscapes [35]. Currently, the majority of existing studies are confined to the analysis of single environmental factors and lack a systematic exploration of their potential interactive effects. Generally, urbanization is accompanied by concurrent changes in vegetation cover and the proportion of impervious surface. This implies that examining only one factor in isolation may not fully reflect the impacts of urbanization on butterfly diversity. In addition, many previous studies have been constrained by relatively short field survey periods and insufficient sampling intensity. Limited field investigations may fail to objectively capture the distribution patterns of urban butterflies. This limitation is especially prominent in tropical regions and developing countries, where research on urban ecosystems is still relatively scarce. These limitations restrict our understanding of the diversity and spatial distribution patterns of urban butterfly communities. In light of these considerations, this study carried out a two-year butterfly survey and monitoring program in Kunming, Yunnan, China, the host city of the Fifteenth Meeting of the Conference of the Parties to the Convention on Biological Diversity (COP15), from 2022 to 2024. Kunming is widely recognized as both a national garden city and a city with abundant biodiversity in China. In recent years, the city has carried out a series of measures for biodiversity conservation. Nevertheless, the absence of long-term monitoring and in-depth research has restricted the scientific formulation of biodiversity conservation policies. This research, taking Kunming as a case study, aims to evaluate the combined impacts of impervious surface and vegetation cover on butterfly species richness, abundance, Shannon index, and Simpson index. High-resolution land-use data were employed to quantitatively evaluate impervious surface and vegetation cover at each sampling site, with the core objective of systematically addressing three key scientific questions. To rigorously answer these questions, high-resolution land-use data were employed to quantitatively characterize impervious surface and vegetation cover at each sampling site, and a multi-model inference framework was adopted for data analysis. The core scientific questions are as follows: (1) How do impervious surface and vegetation cover separately influence the richness and abundance of the butterfly community? (2) Do these two factors show significant interaction effects when influencing the richness and abundance? If such interactions exist, how do they jointly impact the richness and abundance of the butterfly community? (3) How do urban impervious surface and vegetation cover drive the changes in the structure of the butterfly community? Our findings elucidate how urban impervious surface and vegetation cover jointly affect butterfly species richness, abundance, Shannon index, Simpson index, and community structure. This offers a scientific foundation for optimizing urban green space layouts and conserving butterfly biodiversity.

## 2. Materials and Methods

### 2.1. Study Area and Sampling Sites

**Figure 1:**
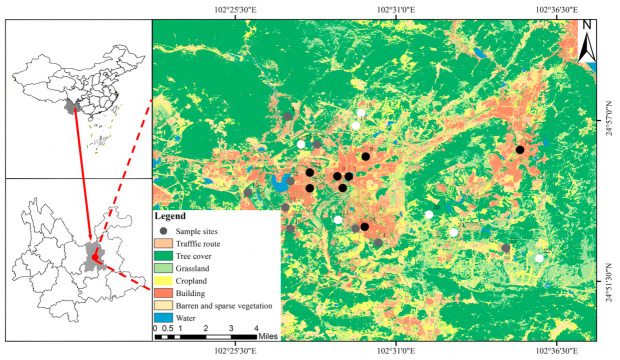
Field investigation area and sampling sites. Note: Black, gray, and white dots indicate high, medium, and low impervious surfaces, respectively. The numbers represent sampling site IDs.

The study area was located in Kunming (102°10′ E to 103°40′ E, 24°23′ N to 26°33′ N), situated in central Yunnan Province, China, covering a total area of approximately 21,012.54 km^2^ (Figure 1). Kunming lies in the Central Yunnan Basin in the central part of the Yunnan-Guizhou Plateau, with an average elevation of about 1891 m. The city has a subtropical plateau monsoon climate characterized by small seasonal temperature variations. The annual mean temperature is approximately 15 °C, with an average temperature of 8.1 °C in January and 20.1 °C in July. The annual precipitation is around 1000 mm, with more than 80% of rainfall occurring during the rainy season from May to October, resulting in distinct wet and dry seasons. Kunming has rich biodiversity and provides an ideal setting for ecological research.

### 2.2. Butterfly Sampling

Butterfly field surveys were conducted once per month from July 2023 to June 2025, totaling 24 sampling events. The surveys used a fixed transect method [36]; we employed a stratified gradient design, and a total of 24 transects were established, each measuring 1000 m in length and 5 m in width. These transects covered habitats across different urbanization gradients, including roadside green belts, residential communities, urban parks, and planted forests. Among the four habitat types, roadside green belts are dominated by grass and shrubs; residential communities are dominated by lawns and ornamental shrubs; urban parks have a complex structure dominated by trees, shrubs, and lawns; and planted forests are dominated by trees. All surveys were conducted under sunny weather conditions with low wind speed and during peak butterfly activity periods. Observers walked along each transect at a speed of 1–2 km/h while conducting the survey during each sampling event. All observers received standardized training in butterfly identification, transect walking protocols, and data recording prior to the field surveys. The observation method was used to record all visible butterflies within 2.5 m on both sides of the transect, 5.0 m above, and 5.0 m ahead. Resting individuals were photographed for documentation, and both species identity and abundance were recorded. Flying individuals were captured using insect nets and placed into triangular paper envelopes for transport back to the laboratory. The identification process relied on “Monograph of Chinese Butterflies” (Volumes 1 and 2), Illustrated Book of “Butterflies of China” (all four volumes), and the National Animal Specimen Resource Center “http://museum.ioz.ac.cn/index.html (accessed on 20 January 2024)”.

### 2.3. Environmental Variable Acquisition

This study calculated impervious surface and vegetation cover based on 1 m high-resolution land cover data which classified the landscape into 11 categories [37]. Among these, seven categories were present in the study area: traffic route, tree cover, cropland, grassland, building, barren land, and water. We used ArcGIS 10.6 to clip the 1 m resolution land cover dataset along the administrative boundary of the study area and to create buffers for each sampling site. Land-use data within each buffer were then extracted to calculate the proportion of impervious surface and vegetation cover at each site. In this study, impervious surfaces consist of industrial, residential, commercial areas, driveways, sidewalks, and other artificial surfaces, excluding barren land with natural rocks. Vegetation cover mainly includes tree cover and grassland. For each sampling site, we created a circular buffer of 500 m radius centered on the midpoint of the transect. Based on the characteristics of urban land use and the distribution of impervious surface in the study area, impervious surface cover was categorized into three levels: low (0–0.1), medium (0.1–0.3), and high (>0.3). Areas with values below 0.1 generally represent rural or semi-natural environments with relatively low impervious surface cover. Areas with values between 0.1 and 0.3 represent zones with a moderate degree of urban development. Areas above 0.3 correspond to typical urban core regions where impervious surfaces dominate the landscape [38]. Based on the vegetation cover characteristics of sampling sites in the study area, vegetation cover was also classified into three levels: low (0–0.3), medium (0.3–0.6), and high (>0.6). Areas with low vegetation cover are mainly characterized by fragmented vegetation and poor habitat connectivity. Areas with medium vegetation cover generally represent semi-natural habitats. Areas with high vegetation cover are typically habitats with relatively continuous vegetation, such as forests and grasslands [39].

### 2.4. Data Analyses

Species richness and abundance were quantified to assess butterfly diversity in the study area [40]. Species richness was defined as the total number of butterfly species recorded during the 24 surveys conducted in the study area, while abundance referred to the total number of butterfly individuals recorded during the same surveys [41]. In addition, the Shannon index and Simpson index were further calculated to comprehensively characterize butterfly community diversity and evenness. First, we employed the R package “vegan” to construct species accumulation curves to evaluate sampling completeness [42]. Second, we used GLMs to examine the combined effects of impervious surface and vegetation cover on butterfly abundance, species richness, Shannon index, and Simpson index. Habitat type was included as a random factor in the models. To evaluate the explanatory power of different variable combinations, a multi-model inference approach was employed for model comparison and selection [43]. Variance inflation factors (VIF < 5) were first calculated for each model, and the results indicated that no multicollinearity existed between impervious surface and vegetation cover [44,45]. We constructed a total of four candidate models for each significant response variable: (i) a model with impervious surface only, to clarify its specific effect on the response variable; (ii) a model with vegetation cover only, to examine its influence on the response variable; (iii) an additive model including both impervious surface and vegetation cover as independent variables, to explain variations in the response variable; and (iv) a model incorporating their interaction term, to evaluate the interactive effects of these two factors on the response variable. The percentage of deviance explained (% dev) was calculated to measure of the explanatory power of each model. All models were ranked based on the Akaike Information Criterion corrected for small sample sizes (AICc) to identify the best-fitting model. Because models with ΔAICc < 2 are considered to have comparable support to the best model [43], model averaging was conducted using the “MuMIn” package to reduce model selection uncertainty [46]. To evaluate the relative importance of variables in the models, the cumulative weights of each variable across all models containing that variable were estimated [43]. In addition, we used the “vegan” package to perform distance-based redundancy analysis (dbRDA) for examining the relationships between impervious surface, vegetation cover, and butterflies’ community composition.

Finally, the “betapart” package was used to calculate beta diversity decomposition of butterfly communities to explore the drivers shaping beta diversity patterns. All statistical analyses were conducted using the statistical software R 4.5.1.

## 3. Results

### 3.1. Butterfly Species Composition

**Figure 2:**
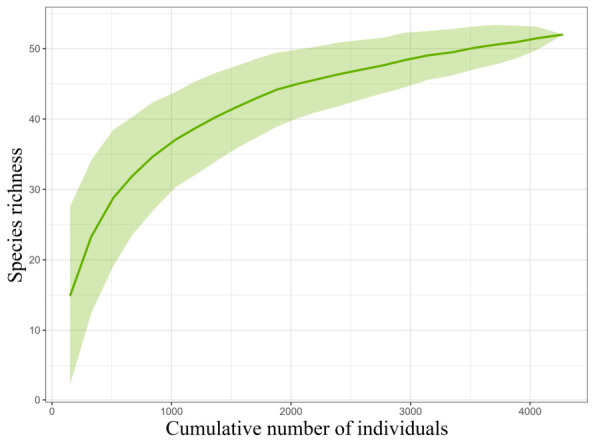
Species accumulation curve based on cumulative number of individuals. The shadow indicates the 95% confidence interval, and the line denotes species richness.

A total of 4276 butterflies were collected in this study, belonging to seven families, 31 genera, and 52 species (Table 1). At the family level, Nymphalidae had the highest number of species, while Hesperiidae, Danaidae, and Satyridae had the fewest. Nymphalidae also had the highest species richness, whereas Satyridae and Hesperiidae had the lowest. In terms of abundance, Pieridae had the highest number of individuals, while Hesperiidae had the lowest. To assess whether the sampling effort was sufficient to represent butterfly species richness in the study area, species accumulation curves were generated to evaluate sampling completeness. The results indicated that the survey data adequately captured the butterfly species richness of the study area (Figure 2).

### 3.2. Impacts of Impervious Surface and Vegetation Cover on Butterfly Species Richness and Abundance

**Figure 3:**
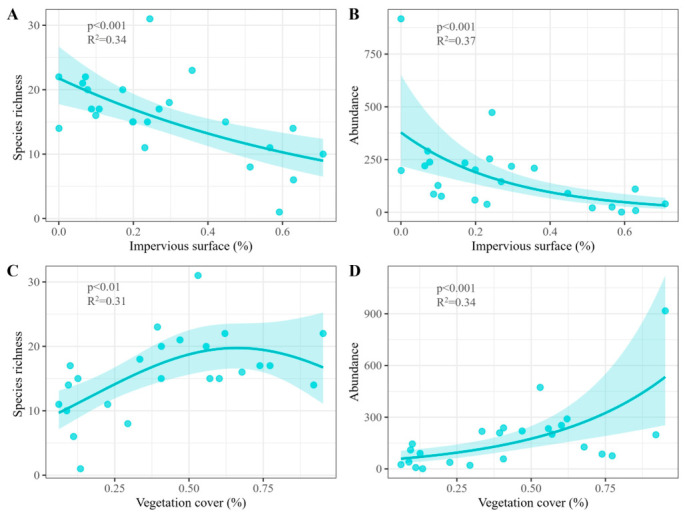
Effects of impervious surfaces and vegetation cover on butterfly species richness and abundance. (**A**) Relationship between impervious surface and butterfly species richness; (**B**) Relationship between impervious surface and butterfly abundance; (**C**) Relationship between vegetation cover and butterfly species richness; (**D**) Relationship between vegetation cover and butterfly abundance. Each point is the observed value from one sampling site. The line denotes the best-fitting regression model, and the shadow indicates the 95% confidence interval.

Regression analysis showed a significant negative relationship between impervious surface and butterfly species richness (R^2^ = 0.34, *p* < 0.001; Figure 3A). Similarly, butterfly abundance also significantly decreased as impervious surface increased (R^2^ = 0.37, *p* < 0.001; Figure 3B). In contrast, vegetation cover exhibited a unimodal relationship with butterfly species richness, with richness first increasing and then decreasing butterfly species richness (R^2^ = 0.31, *p* < 0.01; Figure 3C) and was significantly positively correlated with abundance (R^2^ = 0.34, *p* < 0.001; Figure 3D). However, for the Shannon index and Simpson index, we tested both linear and quadratic terms of impervious surface and vegetation cover, but none of the effects were statistically significant (all *p* > 0.05). Habitat type did not improve model explanatory power and was therefore excluded from the final models for parsimony.

### 3.3. Hypothesis Testing of the Interaction Between Impervious Surface and Vegetation Cover

The model selection results are shown in Table 2. Models with ΔAICc < 2 were considered to have substantial support. For butterfly species richness, the model containing only impervious surface (weight = 0.78) was the best-fitting model. For butterfly abundance, both the model including only impervious surface and the model including only vegetation cover had ΔAICc < 2. However, based on model weights, impervious surface remained the primary factor influencing butterfly abundance (weight = 0.51).

### 3.4. Effects of Impervious Surfaces and Vegetation Cover on Butterfly Community Structure

**Figure 4:**
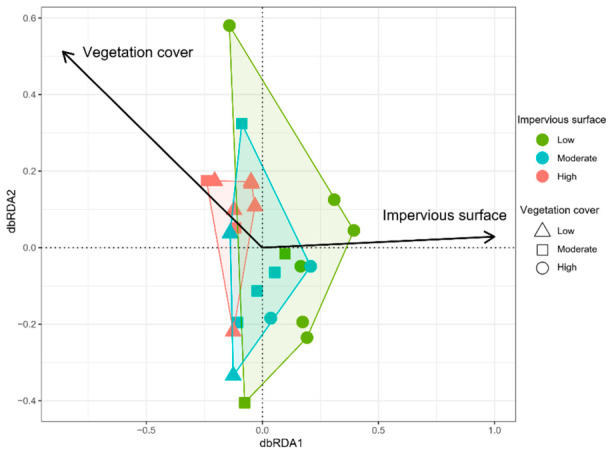
Redundancy analysis of butterfly communities under different degrees of urbanization and vegetation coverage. The colored polygons represent the convex hulls enclosing all sites within each impervious surface category.

Distance-based redundancy analysis (dbRDA) indicated that both impervious surface and vegetation cover had significant effects on butterfly community structure. Along the impervious surface gradient, communities in areas with low impervious surface cover exhibited the highest heterogeneity, followed by communities in moderately impervious areas, while communities in highly impervious areas showed the highest compositional similarity. Along the vegetation cover gradient, communities in areas with low vegetation cover exhibited the greatest compositional similarity, whereas community dissimilarity increased with higher vegetation cover (Figure 4).

### 3.5. Beta Diversity Decomposition of Butterfly Communities in Relation to Impervious Surface and Vegetation Cover

**Figure 5:**
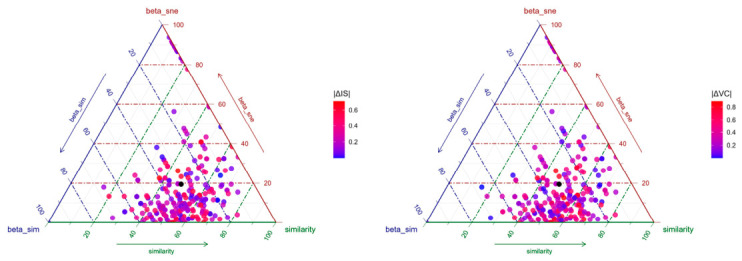
Ternary plot of species turnover, species nestedness, and Sørensen index. Points are colored by absolute differences in impervious surface (|ΔIS|) and vegetation cover (|ΔVC|). Each point corresponds to a pair of sampling sites, representing the relative proportion of the three components for their pairwise beta diversity. The color gradient ranges from blue to red, with redder colors indicating greater absolute differences in the target environmental variable between paired sites. The black solid dot indicates the overall mean value of the three components across all pairwise site comparisons.

To further determine how impervious surface and vegetation cover drive butterfly community structure, beta diversity was decomposed into species turnover and nestedness components. Based on the Sørensen index, the decomposition of butterfly community beta diversity showed a mean dissimilarity of 0.45, with species turnover averaging 0.416 and nestedness averaging 0.034 (Figure 5). Sampling sites were primarily distributed within the species turnover component, indicating that butterfly community structure was mainly driven by species turnover, while the contribution of nestedness was limited.

## 4. Discussion

In the context of rapid urbanization, the impact of urban expansion on biodiversity has emerged as a crucial concern in ecological research [47]. The expansion of impervious surfaces due to urbanization not only alters the land-use pattern but also results in habitat loss and fragmentation, significantly affecting the diversity of butterflies and other species. Meanwhile, as an important ecological indicator, the influence of vegetation cover on biodiversity is also substantial [2,23,48]. We conducted a systematic analysis of the impacts of impervious surfaces and vegetation cover on butterfly species richness, abundance, Shannon index, and Simpson index. The findings demonstrated that impervious surface had a significant negative correlation with both the richness and abundance of butterfly species. In contrast, vegetation cover exhibited a unimodal relationship with butterfly species richness, with richness first increasing and then decreasing butterfly species richness and was significantly positively correlated with abundance. The Shannon and Simpson indices were not significantly influenced by either environmental factor. Multi-model inference suggested that impervious surface was the primary environmental factor influencing butterfly species richness, while the positive influence of vegetation cover was relatively feeble. Regarding species abundance, impervious surface remained the key factor, and vegetation cover had a weaker impact compared to impervious surface. Since the cumulative weight of the interaction term in the candidate models was low, the effects of impervious surface and vegetation cover on butterfly species richness and abundance were mainly manifested as independent main effects. Distance-based redundancy analysis (dbRDA) showed that butterfly community heterogeneity was highest in areas with low impervious surface and high vegetation cover, whereas community composition was more homogeneous in areas with high impervious surfaces and low vegetation cover. Beta diversity decomposition further revealed that the differences among butterfly communities were primarily driven by species turnover. These findings offer empirical evidence for comprehending the mechanisms by which urbanization impacts biodiversity and guide urban ecological restoration efforts.

As expected from previous studies, this study found that both butterfly species richness and abundance declined with increasing impervious surface, consistent with some earlier findings [26,49]. However, it is important to note that this trend shows variations across different studies. In our research, species richness decreased monotonically as the impervious surface increased, whereas the abundance did not display the previously reported unimodal pattern with higher values in highly urbanized areas [13]. Compared with Blair and Launer, who discovered that the richness of butterfly species along the urbanization gradient showed a unimodal distribution, while the abundance decreased continuously as the urbanization intensity increased, our study did not observe a unimodal pattern for either richness or abundance. This disparity may be ascribed to the spatial scale [50]. Unimodal patterns are more likely to be apparent in large-scale analyses. In contrast, at local scales, the negative impacts of habitat loss and microclimatic degradation caused by impervious surfaces within cities may overshadow the buffering effect of suburban transition zones [51,52]. Impervious surfaces generally exhibit higher surface temperatures, which lead to drier, more extreme, and unstable microclimatic conditions that are unfavorable for larval development, adult butterfly activity, and foraging [53]. The increase of impervious surface will aggravate landscape fragmentation, form a physical barrier for butterfly diffusion, greatly reduce the connectivity between patches, and hinder the cross-patch migration and population exchange of butterflies [54]. Moreover, the road networks linked to impervious surfaces elevate the direct mortality risk during butterfly movement, rendering it challenging for species with limited dispersal ability to sustain stable populations in urban environments [55]. Meanwhile, our study confirmed that vegetation cover exhibited a unimodal relationship with butterfly species richness, and was significantly positively correlated with butterfly abundance. Moreover, the effect on abundance was stronger than that on richness. This difference indicates that butterfly abundance is more sensitive to vegetation cover. High vegetation cover typically corresponds to larger and more connected habitat patches, which facilitate butterfly dispersal, colonization, and the maintenance of stable populations across fragmented landscapes [56]. An increase in vegetation cover enhances the availability of nectar resources, which can rapidly support the population expansion of generalist and widely distributed species. Continuous vegetation also offers refuges that enable butterflies to avoid the heat island effects and human disturbances [28]. Furthermore, the widespread fragmentation and low resource heterogeneity of urban green spaces directly increase interspecific competition among butterfly populations, which initially causes declines in abundance [57,58,59]. In contrast, species richness may not increase continuously with vegetation cover, but instead reach a peak at intermediate levels of vegetation cover. At low vegetation cover, butterfly richness is constrained by limited habitat area, poor resource availability, strong edge effects, high temperatures, and frequent human disturbance, as well as by the low proportion of native host plants in urban artificial greening, which limits the availability of larval host plants and thus fails to support specialist butterfly species [60]. However, excessively high vegetation cover may reduce butterfly richness by decreasing habitat heterogeneity [59]. Dense or closed-canopy vegetation limits sunlight availability and reduces open or semi-open microhabitats, thereby decreasing the species richness of sun-loving butterflies and large butterfly species that prefer such habitats [59,61]. Therefore, intermediate vegetation cover may provide a more suitable balance between habitat connectivity, resource availability, microclimatic buffering, and habitat heterogeneity, resulting in a unimodal pattern of butterfly species richness [62]. The absence of significant effects of impervious surface and vegetation cover on the Shannon and Simpson indices may be attributed to the fact that, although these factors altered the number of rare species and total individual abundance, they did not significantly change the community relative abundance pattern. Urban butterfly communities are often dominated by a few generalist species that are highly tolerant to habitat fragmentation and vegetation changes, with their dominance remaining relatively stable; thus, the Simpson index shows little response. Meanwhile, rare or specialist species, despite possible increases or decreases along the gradient, have limited influence on the Shannon index due to their low individual numbers and high spatial variability. The negligible explanatory contribution of habitat type may indicate that, in heterogeneous urban landscapes, the internal composition of transects, particularly vegetation cover and impervious surface, better captures the habitat gradients relevant to butterfly communities than broad habitat-type classifications.

The multi-model inference results clearly indicated that impervious surface was the key driver behind the declines in butterfly species richness. This finding suggests that in urban settings, impervious surface may serve as a more direct limiting factor compared to only consider the vegetation cover at the landscape scale. A plausible explanation is that even when vegetation resources are available, butterflies with limited dispersal capabilities are unable to traverse impervious surface to access suitable habitats, which ultimately results in a reduction in species richness [62]. Unlike species richness, which was mainly affected by impervious surface, butterfly abundance was influenced by both impervious surface and vegetation cover. The impervious surface served as the primary driver, while the influence of pure vegetation cover was relatively limited. This could be due to the fact that a higher proportion of impervious surface not only reduces suitable habitats for butterflies but can also disrupt certain butterfly behaviors, such as mud-puddling. Mud-puddling is mainly carried out by male butterflies to obtain minerals and water from moist soil, detritus, or feces to meet their metabolic needs [63,64]. In highly impervious urban areas, suitable mud-puddling substrates are frequently scarce. This scarcity restricts the nutrient intake of male butterflies, which in turn may impact female reproductive success and limit population maintenance. By comparison, vegetation cover exerts a relatively weaker influence, implying that its beneficial effects only manifest in habitats not heavily degraded by impervious surfaces. Once impervious surfaces undergo extensive expansion, localized increases in vegetation are insufficient to counterbalance the adverse consequences of widespread habitat modification [25]. Furthermore, our results demonstrate that high vegetation coverage alone does not necessarily promote butterfly species richness and abundance. Most urban green spaces are predominantly composed of exotic ornamental plants, which provide limited ecological support for butterfly populations. This may explain why impervious surfaces emerge as the dominant driving factor [14,60,65,66]. Notably, no interactive effect between impervious surface and vegetation cover was identified in this study, signifying that their influences on butterfly abundance operate independently and exert additive effects overall. Consistent with our findings, Fontana et al. reported independent and additive impacts of building cover and tree cover on urban bird communities [67]. However, this result diverges from previous research on Hymenopteran pollinators, in which a significant interactive effect between impervious surface and vegetation cover on pollinator abundance was detected [68]. These interspecific discrepancies are likely attributable to taxonomic differences in ecological niche demands, dispersal capacity, and resource exploitation strategies among insect groups [69].

Distance-based redundancy analysis (dbRDA) revealed that along the impervious surface gradient, the butterfly communities demonstrated an increasing tendency toward homogenization as the impervious surface area increased. Along the vegetation cover gradient, regions with higher vegetation cover displayed greater dissimilarity among butterfly communities. Beta diversity decomposition further revealed that the differences among butterfly communities were primarily driven by species turnover under the environmental filtering effects of impervious surface and vegetation cover. In areas characterized by moderate-to-high impervious surface and low vegetation cover, habitat deterioration and the reduction in niche availability intensified environmental filtering. As a result, numerous species that depend on stable habitats were eliminated, leaving only a small number of highly adaptable species possessing strong dispersal ability and a wide range of host-plant utilization, which led to community homogenization [70]. In contrast, areas with low impervious surface and high vegetation cover offer more heterogeneous habitats that support the coexistence of butterfly species with distinct ecological niches, thus maintaining higher beta diversity [71,72].

Our results elucidate the driving mechanisms of impervious surface and vegetation cover on urban butterfly species richness and abundance and stress that regulating the extent of impervious surface, preserving large-scale vegetation, and optimizing vegetation configuration could be effective measures to alleviate the negative impacts of urbanization on butterfly diversity. Similar urbanization effects are probably prevalent in tropical and subtropical regions, including Kunming. Therefore, these findings provide a scientific basis for biodiversity conservation in these areas. In particular, the results hold significant practical implications for promoting the development of urban green spaces and restoring ecological functions. However, this study does have certain limitations. First, vegetation cover was used as the sole vegetation indicator, failing to capture fine-scale habitat quality attributes such as plant species composition and nectar resource availability, which could lead to an underestimation of the true effects of vegetation-related factors. Second, this study relied exclusively on daytime transect counts without employing fruit-baited traps or crepuscular surveys. Such sampling limitations may result in an underestimation of the diversity of saprophagous Nymphalidae and crepuscular butterflies, and also reduce the survey efficiency for canopy-dwelling butterflies in forest patches. Third, habitat patch connectivity was not directly quantified. Consequently, it cannot be disentangled whether the significant effects of impervious surfaces are driven by habitat loss, patch isolation, or direct local environmental effects. Future studies are recommended to integrate multi-scale landscape metrics, fine-scale vegetation surveys, and complementary sampling approaches to validate and extend the findings of this study.

## 5. Conclusions

Our study conducted a systematic evaluation of the effects of impervious surfaces and vegetation cover on urban butterfly species richness, abundance, Shannon index, Simpson index, and community structure. The results indicated that impervious surface was the crucial factor affecting butterfly species richness. In terms of butterfly abundance, impervious surface served as the primary driving factor, whereas the influence of vegetation cover was comparatively limited, and the two mainly demonstrated independent additive main effects. Notably, the Shannon and Simpson indices were not significantly influenced by either environmental factor. The differences among butterfly communities primarily stemmed from species turnover driven by environmental filtering through impervious surface and vegetation cover. Butterfly communities tend to become homogenized in areas with high impervious surface and low vegetation cover. Our findings offer empirical evidence for comprehending butterfly diversity dynamics under urbanization in tropical and subtropical regions, including Kunming, and provide scientific support for urban ecosystem management and species conservation strategies.

## Figures and Tables

**Table 1:** Butterfly species composition in Kunming.

Family	Number of Genera	Proportion	Number of Species	Proportion	Number of Individuals	Proportion
Pieridae	4	12.90%	9	17.31%	2418	56.55%
Lycaenidae	8	25.81%	10	19.23%	1257	29.40%
Hesperiidae	2	6.45%	3	5.77%	8	0.19%
Nymphalidae	10	32.26%	13	25.00%	265	6.20%
Satyridae	2	6.45%	3	5.77%	115	2.69%
Danaidae	2	6.45%	4	7.69%	58	1.35%
Papilionidae	3	9.68%	10	19.23%	155	3.62%
Total	31	100.00%	52	100.00%	4276	100.00%

**Table 2:** Multi-model inference based on species richness and abundance.

	Model	AICc	∆AICc	Weight	R^2^
Richness	Impervious surface	155.05	0.00	0.778	0.34
	Vegetation cover	159.27	4.22	0.094	0.31
	Impervious surface + Vegetation cover	159.08	4.03	0.104	0.39
	Impervious surface × Vegetation cover	162.03	6.98	0.024	0.40
Abundance	Impervious surface	291.55	0.00	0.513	0.37
	Vegetation cover	292.94	1.39	0.256	0.34
	Impervious surface + Vegetation cover	293.57	2.02	0.187	0.39
	Impervious surface × Vegetation cover	296.48	4.93	0.044	0.40

Note: ΔAICc values less than 2 means the model is effective.

## Data Availability

The original contributions presented in this study are included in the article/Supplementary Materials. Further inquiries can be directed to the corresponding author.

## Supplementary Materials

The following supporting information can be downloaded at: https://www.mdpi.com/article/10.3390/insects17050482/s1, Table S1: Sampling site information. Table S2: Butterfly species checklist.
