# Supplementary material for: Urban Expansion and Butterfly Diversity: The Synergistic Effects of Impervious Surface and Vegetation Cover
Source: Insects. 2026 May 8;17(5):482. doi: 10.3390/insects17050482 (PMC13207098; doi:10.3390/insects17050482)
Supplement: Supplementary file 1 [file insects-17-00482-s001.zip › insects-4254073-supplementary.pdf]

Supplementary data

Table S1. Sampling site information

Sampling site	Longitude (N)	Latitude (E)	Richness	Abundance	Impervious surface / Rank	Vegetation coverage / Rank
S1	102.455	24.956	17	76	0.108403051 / 1	0.772826249 / H
S2	102.457	24.919	17	145	0.268971643 / 2	0.100070962 / L
S3	102.49	24.922	15	90	0.447335441 / 3	0.126223463 / L
S4	102.507	24.884	15	253	0.237762559 / 2	0.60204983 / H
S5	102.494	24.892	11	38	0.230791415 / 2	0.226296808 / L
S6	102.472	24.94	15	201	0.199335445 / 2	0.570355855 / H
S7	102.468	24.915	1	1	0.591655465 / 3	0.134680433 / L
S8	102.5	24.933	11	25	0.566119672 / 3	0.061793443 / L
S9	102.484	24.922	6	8	0.629487398 / 3	0.111477109 / L
S10	102.433	24.913	20	234	0.171511962 / 2	0.557905783 / M
S11	102.484	24.897	17	86	0.087641921 / 1	0.738716849 / H
S12	102.487	24.915	23	209	0.35763535 / 3	0.393841778 / M
S13	102.58	24.881	15	58	0.198372326 / 2	0.406574281 / M
S14	102.588	24.937	14	110	0.628271333 / 3	0.094062912 / L
S15	102.463	24.94	16	127	0.09924397 / 1	0.678411917 / H
S16	102.457	24.892	31	473	0.244252878 / 2	0.530410128 / M
S17	102.454	24.904	18	218	0.296427764 / 2	0.334564065 / M
S18	102.494	24.951	21	220	0.063970024 / 1	0.46965745 / M
S19	102.497	24.958	20	238	0.07724471 / 1	0.407131365 / M
S20	102.536	24.9	14	198	0.000532466 / 1	0.919925357 / H
S21	102.55	24.89	22	290	0.071622618 / 1	0.620920516 / H
S22	102.599	24.875	22	917	0.0001 / 1	0.951479932 / H
S23	102.468	24.924	8	21	0.513217378 / 3	0.29398078 / M
S24	102.499	24.893	10	40	0.70890695 / 3	0.08844821 / L

Note: The numbers 1, 2 and 3 indicate low, medium and high levels of impervious surface, while L, M and H represent low, medium and high levels of vegetation coverage, respectively.

Table S2. Butterfly species checklist

Number	Family	Genus	Species
1	Pieridae	*Pieris*	*Pieris rapae*
2			*Pieris canidia*
3			*Pieris napi*
4			*Pieris melete*
5			*Pontia edusa*
6		*Colias*	*Colias poliographus*
7			*Colias fieldii Ménétriés*
8		*Eurema*	*Eurema mandarina*
9		*Gonepteryx*	*Gonepteryx amintha*
10	Lycaenidae	*Zizina*	*Zizina otis*
11		*Leptotes*	*Leptotes plinius*
12		*Udara*	*Udara dilecta*
13		*Jamides*	*Jamides bochus*
14			*Jamides alecto*
15		*Celastrina*	*Celastrina oreas*
16		*Chilades*	*Chilades pandava*
17		*Lampides*	*Lampides boeticus*
18		*Acytolepis*	*Acytolepis puspa*
19		*Everes*	*Everes argiades*
20	Hesperiidae	*Polytremis*	*Polytremis discreta*
21		*Parnara*	*Parnara guttata*
22			sp1
23	Nymphalidae	*Argynnis*	*Argynnis hyperbius*
24		*Vanessa*	*Vanessa cardui*
25			*Vanessa indica*
26		*Araschnia*	*Araschnia prorsoides*
27		*Symbrenthia*	*Symbrenthia lilaea*
28		*Junonia*	*Junonia orithya*
29			*Junonia hierta*
30		*Neptis*	*Neptis hylas*
31		*Polyura*	*Polyura athamas*
32		*Phalanta*	*Phalanta phalantha*
33		*Aglais*	*Aglais urticae*
34		*Calinaga*	*Calinaga davidis*
35			sp1
36	Satyridae	*Ypthima*	sp1
37			*Ypthima nareda*
38		*Mycalesis*	*Mycalesis francisca*
39	Danaidae	*Parantica*	*Parantica sita*
40		*Danaus*	*Danaus genutia*
41			*Danaus chrysippus*
42			sp1
43	Papilionidae	*Papilio*	*Papilio bianor*
44			*Papilio maackii*

**Number**	**Family**	**Genus**	**Species**
45			*Papilio dialis*
46			*Papilio xuthus*
47			*Papilio helenus*
48			sp1
49		*Byasa*	sp1
50			*Byasa dasarada*
51		*Graphium*	*Graphium sarpedon*
52			*Graphium cloanthus*
